# Subtle Ecological Gradient in the Tropics Triggers High Species-Turnover in a Local Geographical Scale

**DOI:** 10.1371/journal.pone.0156840

**Published:** 2016-06-08

**Authors:** Dinh T. Nguyen, Jesús Gómez-Zurita

**Affiliations:** 1 Animal Biodiversity and Evolution, Institute of Evolutionary Biology (CSIC-Universitat Pompeu Fabra), Barcelona, Spain; 2 Institute of Ecology and Biological Resources, Vietnam Academy of Science and Technology (VAST), 18 Hoang Quoc Viet, Ha Noi, Vietnam; National Cheng-Kung University, TAIWAN

## Abstract

Our perception of diversity, including both alpha- and beta-diversity components, depends on spatial scale. Studies of spatial variation of the latter are just starting, with a paucity of research on beta-diversity patterns at smaller scales. Understanding these patterns and the processes shaping the distribution of diversity is critical to describe this diversity, but it is paramount in conservation too. Here, we investigate the diversity and structure of a tropical community of herbivorous beetles at a reduced local scale of some 10 km^2^, evaluating the effect of a small, gradual ecological change on this structure. We sampled leaf beetles in the Núi Chúa National Park (S Vietnam), studying changes in alpha- and beta-diversity across an elevation gradient up to 500 m, encompassing the ecotone between critically endangered lowland dry deciduous forest and mixed evergreen forest at higher elevations. Leaf beetle diversity was assessed using several molecular tree-based species delimitation approaches (with mtDNA *cox1* data), species richness using rarefaction and incidence-based diversity indexes, and beta-diversity was investigated decomposing the contribution of species turnover and nestedness. We documented 155 species in the area explored and species-richness estimates 1.5–2.0x higher. Species diversity was similar in both forest types and changes in alpha-diversity along the elevation gradient showed an expected local increase of diversity in the ecotone. Beta-diversity was high among forest paths (average Sørensen's dissimilarity = 0.694) and, tentatively fixing at 300 m the boundary between otherwise continuous biomes, demonstrated similarly high beta-diversity (Sørensen's dissimilarity = 0.581), with samples clustering according to biome/elevation. Highly relevant considering the local scale of the study, beta-diversity had a high contribution of species replacement among locales (54.8%) and between biomes (79.6%), suggesting environmental heterogeneity as the dominant force shaping diversity at such small scale, directly and indirectly on the plant communities. Protection actions in the Park, especially these addressed at the imperative conservation of dry forest, must ponder the small scale at which processes shape species diversity and community structure for inconspicuous, yet extraordinarily diverse organisms such as the leaf beetles.

## Introduction

Beta-diversity describes the changes in species composition across local samples in a particular region [1], and it has been recognised as a pivotal topic in community ecology with current attention mostly centred on its properties and value to define patterns and processes of species assemblages [2–4]. Indeed, the study of beta-diversity is interesting as a descriptive measure of the structure of biodiversity. However, its main advantage stems precisely from its potential to untangle the specific processes that shape diversity, establishing a link between these descriptive patterns of diversity and explanations of their origin when combined with information on spatial heterogeneity [3]. Despite the long history and uncontested importance of this concept, the revisionary work by C. Rahbek already identified the paucity of studies on species richness along environmental gradients considering the combined effects of both alpha- and beta-diversity [5]. And one decade later, there is a perception that our understanding of beta-diversity still demands more work (e.g., [6]). Thus, it is generally recognised that combined analyses of species turnover and environmental conditions are starting (e.g., [7]) or in their infancy (e.g., [8]). Several causes are considered when interpreting beta-diversity patterns, including biotic and abiotic factors. Legendre and coauthors [2], in their analysis of explanations for beta-diversity, identified three competing hypotheses or factors emphasising the causes for the observed patterns: (1) communities assemble in response to antagonistic biological interactions; (2) the history of dispersal (and speciation) determines the composition of communities; and (3) the species present in a given area are selected by environmental conditions, when they are heterogeneous across the landscape. Which specific factors should be regarded as being the potential drivers of spatial structuring of communities have to do with two important aspects that have demanded considerable attention: spatial scale and environmental gradients.

It is understood that scale, measured as grain (size of local sample) and extent (spatial coverage of local samples), is important and has an influence on beta-diversity, but the cause is not fully understood [5, 9–12]. In general, scale is considered important because any of the aforementioned factors—but mainly history and landscape heterogeneity—and thus the processes that determine biodiversity patterns from local to global, is expected to vary when considering areas of different size ([10] and references therein; [13]). In part related to this, scale is also relevant depending on the organism or group of organisms investigated and the considerations one can draw from beta-diversity estimates. Perhaps because of the multiple interacting factors and the multiple ways in which they can interact, the impact of spatial scale on turnover is contentious, without resulting in a clear emergent pattern. For example, most studies have reported higher beta-diversity at smaller compared to larger scales (e.g., [7, 11, 14]), but the pattern is not unique (see [13]). Different spatial scale perceptions may depend on the taxonomic group, in part by the effect of species range size on beta-diversity estimates, so that restricted ranges relative to sampling scale can contribute to high diversity (e.g., [13]).

**Fig 1:**
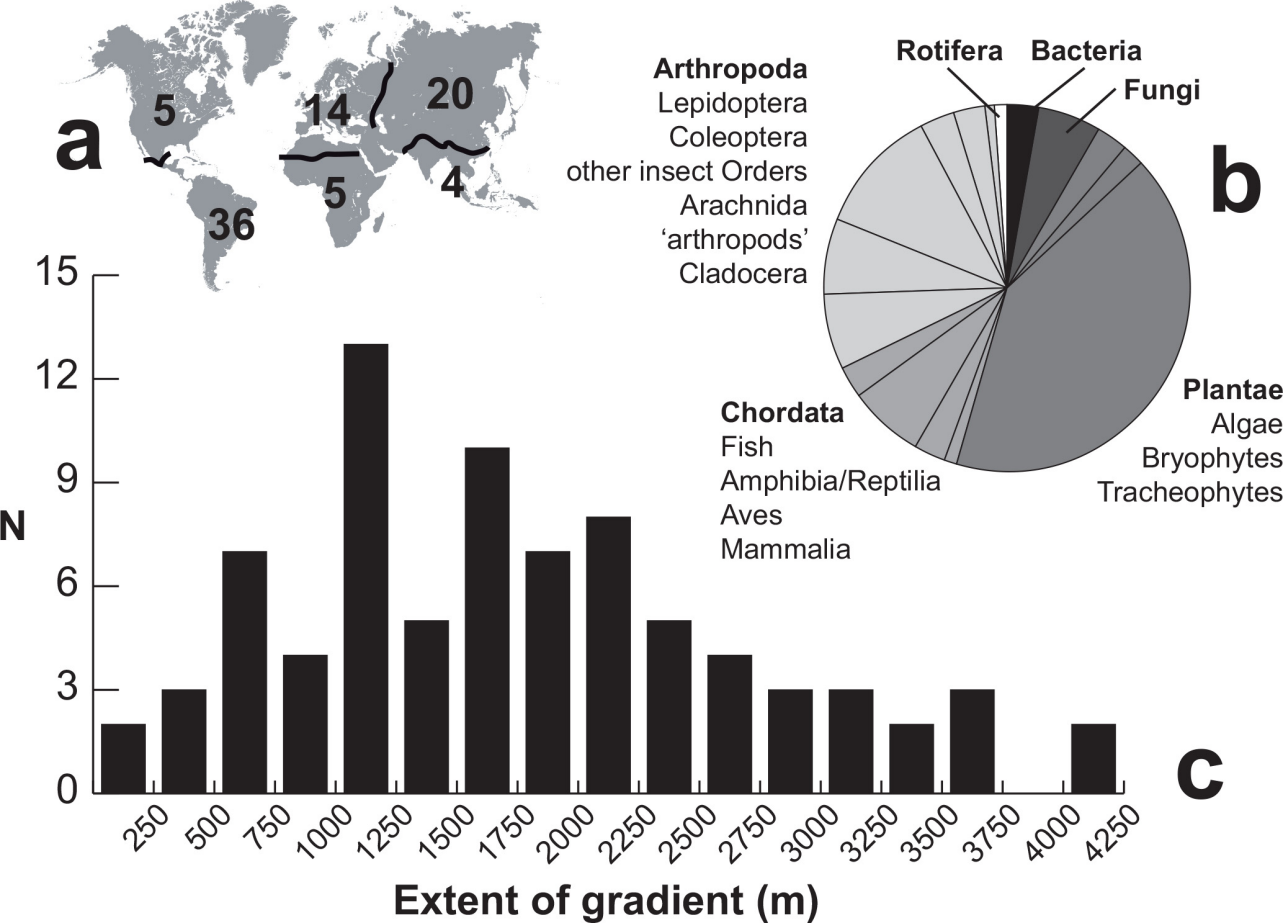
Graphical summary of representative studies focusing on beta-diversity along elevation gradients. The list of representative studies is parsed in the different panels highlighting their biogeographic domain (a), their taxonomic focus (b) and the extent of the studied gradient (c).

Relevant for the purposes of the current study, while knowledge on species assemblages or species distributions is vast, particularly in the botanical literature, fine-scale beta-diversity over a continuous gradient is seldom examined [15]. At these smaller scales, one of the classical approaches used by community ecologists to investigate the factors acting on community assemblage exploit elevation as surrogate measure of environmental gradients [8, 12]. Elevation gradients usually involve environmental (e.g. temperature, atmospheric pressure, precipitation, radiation, or substrate composition, among several other factors) and concomitant ecological changes, which act as potential factors leading to increased local and regional diversity as well as community changes along the gradient [8, 16]. There is a rich literature that demonstrates changes in species assemblages along elevation gradients (e.g., 81 articles including the terms 'beta diversity' and either 'elevation* gradient' or 'altitud* gradient' and filtered for analysis of community changes along continuous gradients were retrieved from a search in Web of Science Core Collection, Thomson Reuters Ltd.; 17 August 2015). The number of studies in tropical or temperate (or even sub-arctic) regions is well balanced, with most research being carried out in elevation gradients in the Neotropics, followed by studies in the eastern Palaearctic, mainly in China (Fig 1). In any case, the field is clearly dominated by the study of plant communities, and mainly focusing on vascular plants (41.5% of representative literature; e.g., [17–19]). The analysis of species turnover along altitudinal gradients in animals exploits some species-rich groups of arthropods, particularly insects (24.5%), mostly beetles and butterflies (e.g., [20–23]), or some emblematic groups of vertebrates, mainly birds (6.6%; e.g., [15, 24]). Soil community ecologists have paid attention to species turnover in elevation gradients for bacteria (2.8%; e.g., [25–26]) and fungi (5.7%; e.g., [27]). These and many other studies typically investigated extensive elevation changes (more than 80% of the representative studies investigated gradient extents beyond 1000 m; Fig 1), and largely confirmed that species diversity and assemblages change with elevation.

In the case of tropical montane landscapes, faunas can be particularly diverse, with high local alpha-diversity and high species turnover between zones or habitats [15, 23, 28–29]. However, the actual diversity patterns associated with these gradients and the mechanisms responsible for them still generate some debate [5, 8, 12, 30], and they can vary depending on taxa, scale, or geographic region, among others [8]. From the specific angle of beta-diversity, some studies report low species turnover in lowlands compared to highlands, with higher diversity in the latter (e.g., Costa Rican scarab beetles in [23]), while others find higher pattern diversity and species richness at lower elevations (e.g., Costa Rican birds in [15]), highlighting again taxon-specific responses to environmental gradients [31].

We are interested in the study of insect diversity and community changes across environmental gradients at very small, local scales, which can be nevertheless relevant for management decisions affecting these organisms. In particular, we examine a continuous ecological transition from dry tropical deciduous forest to moist deciduous and semi-evergreen seasonal forests across a short elevation gradient in a protected area in southern Vietnam, the Núi Chúa National Park. To investigate the effect and strength of biome transitions at very reduced scales, we address this study to a taxonomic group, the leaf beetles (Chrysomelidae), for which we have no previous knowledge on their diversity but we expect them to be highly diverse based on partial regional catalogues in nearby areas (e.g., [32–35]). Moreover, their tight association with plants allows predicting that heterogeneity in plant assemblages can potentially drive changes in leaf beetle communities that use them as primary resources. Thus, our specific goals are (1) characterising the community of leaf beetles in the Núi Chúa National Park (alpha-diversity), and (2) evaluating the structure of leaf beetle communities and spatial changes across samples and elevation (beta-diversity). A combined consideration of these two components of diversity on a system never investigated before shall provide us with insight for further research on the causes shaping leaf beetle assemblages in the tropics and the scales relevant for similar studies. Beyond descriptive and scientific goals, our study is strongly motivated by conservation needs. We centred our work on the Núi Chúa National Park because it represents one of the bastions of preservation of the highly endangered seasonally dry tropical forest in South East Asia [36]. These endangered forests still present a relatively healthy condition in Núi Chúa, forming a non-fragmented habitat stripe in coastal lowland areas with a continuous succession to moister forest types in the elevation gradient, up to rainforest in the highest peak (1039 m a.s.l.). Our results can contribute to raising awareness on the value of integral conservation to preserve diversity and processes occurring at very local scales.

## Materials and Methods

### Study site and sampling strategy

**Fig 2:**
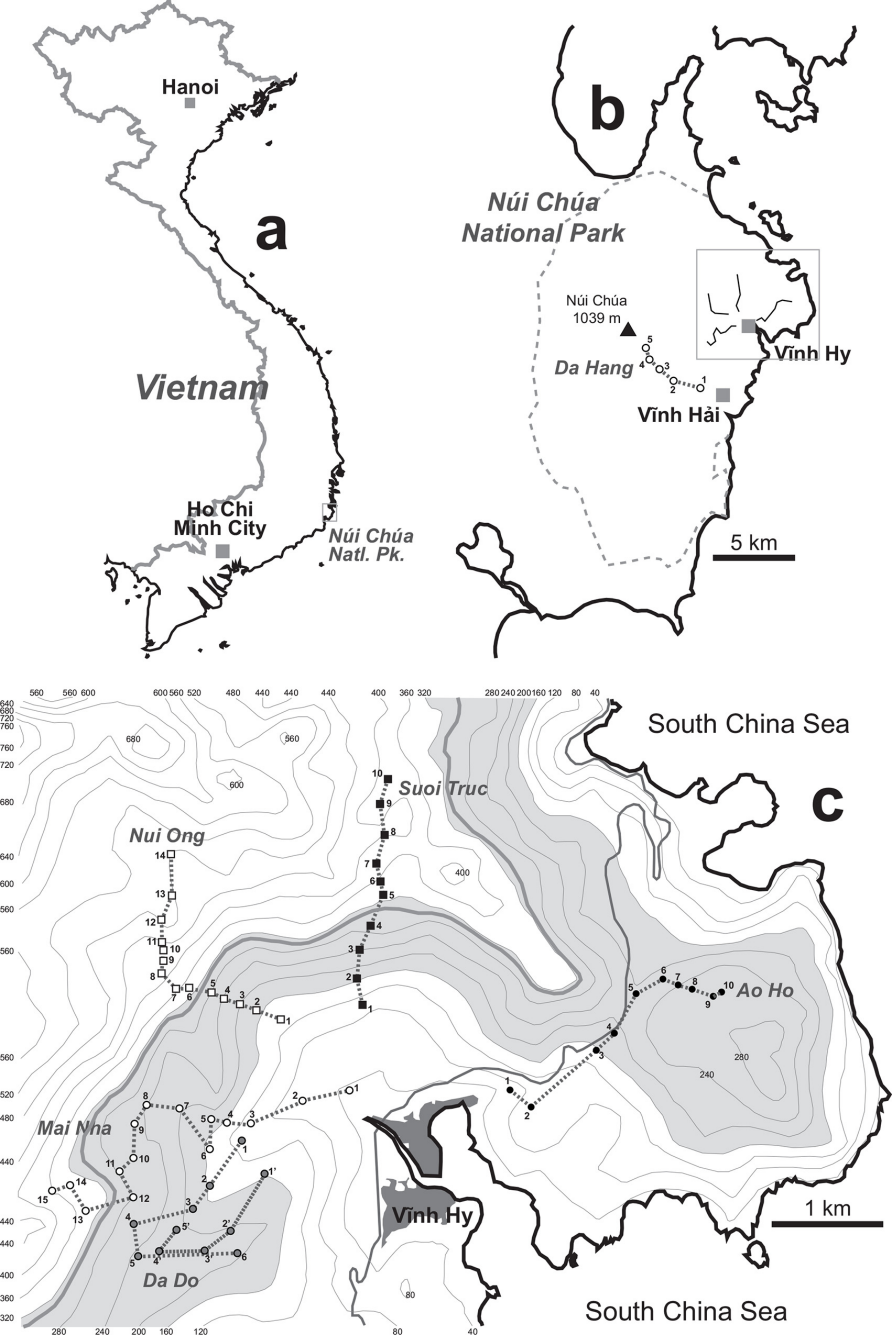
Sampling of Chrysomelidae in the Núi Chúa National Park. (a) Geographic location of the study site in southern Vietnam and (b) placement and general sampling design in the Núi Chúa National Park. (c) Forest paths sampled from Vinh Hy and into higher elevations across the putative ecotone (gray area) between seasonally dry tropical forest in coastal lowlands and moister forest types. A thick gray line marks a pragmatic boundary between biome types putatively representing the strongest structuring effect of the ecotone (see main text for details).

The study was conducted in the Núi Chúa National Park (11°43.67'N 109°11.36'E; Ninh Hai district, Ninh Thuan province, South Vietnam). This location is in the dry tropics, dominated by dry and hot climate at low elevations (<600 m a.s.l.) and moister conditions as elevation increases. The orography and climatic conditions favour ecological gradients at a very reduced, local scale, including a floristic transition from relatively well-preserved restored sclerophyll deciduous forest and scrubland at low elevations to primeval broadleaf and evergreen forest at higher elevation. We sampled along five forest paths with moderate elevation gradients (from sea level, up to 490 m a.s.l.) and more or less radially from the coastal locality of Vinh Hy, and an additional path (Da Hang), 8–10 km southwest from Vinh Hy, from the small settlement of Dinh Bà up to 775 m a.s.l. in the Núi Chúa mountain (Table 1; Fig 2). The studied elevation gradient, particularly in forest paths reaching higher elevations, was concomitant with a change in plant community, as assessed independently by a botanist, Mr. Nguyen Hung Manh (Institute of Ecology and Biological Resources, Hanoi, Vietnam). Each path started in seasonally dry forest and reached in most cases evergreen forest or a transitional area, except in the case of the Da Hang, which penetrated deeply into rainforest. Da Hang (nearly 4.5 km) was sampled only once in early May 2012 and, due to severe difficulties for fieldwork, discarded for community analyses. By exclusion of Da Hang, linear length of these paths ranged between slightly over 2 km for Nui Ong and about 3.5 km for Mai Nha. The longest distance between any two sampling points was about 5.6 km (between Ao Ho and Mai Nha) and the prospected area covered an approximate surface of 10 km^2^. Three paths reaching 407–490 m a.s.l. were traversed ten times, once during June, July and September (2012) and January (2013) and twice monthly in February-April (2013). Da Do and Ao Ho, the paths reaching lower elevations (244 and 261 m a.s.l., respectively) and relatively uniform in plant community, were visited only four times, in June, July and September (2012) and January (2013). Specimens of Chrysomelidae were collected around the same spots along these forest paths every time, devoting approximately 10 min to beat low tree branches and understorey vegetation, up to arm reach, catching the fallen beetles from a beating tray. Specimens were immediately stored in absolute ethanol for DNA preservation and collecting area georeferenced for subsequent analyses. Samples were collected and exported to the lab for processing under permits no. 1251/SNNPTNT-VP (27/Jul/2012) issued by Ninh Thuan Office of Agriculture and Rural Development and 3678/UBND-NV (3/Aug/2012) issued by Ninh Thuan People's Committee (Vietnam).

**Table 1:** Sampling of Chrysomelidae in Núi Chúa National Park.

Locality	Latitude	Longitude	Elev.	N	Locality	Latitude	Longitude	Elev.	N
**DH1**	11.6956389	109.162736	109	11	**MN7**	11.7251667	109.177236	180	9
**DH2**	11.7021667	109.148444	233	15	**MN8**	11.7251806	109.175125	224	15
**DH3**	11.7096528	109.141736	506	2	**MN9**	11.7238611	109.174472	244	14
**DH4**	11.7156111	109.136944	661	13	**MN10**	11.7216528	109.174681	258	9
**DH5**	11.7221667	109.136111	714	9	**MN11**	11.7207500	109.173917	269	3
					**MN12**	11.7192361	109.174944	284	15
**AH1**	11.7279722	109.202944	50	3	**MN13**	11.7182222	109.172222	324	3
**AH2**	11.7266389	109.204750	82	6	**MN14**	11.7196667	109.171222	358	3
**AH3**	11.7310278	109.209889	142	2	**MN15**	11.7192500	109.170222	364	4
**AH4**	11.7322222	109.211278	173	2					
**AH5**	11.7352778	109.212972	200	4	**NO1**	11.7334444	109.184389	122	15
**AH6**	11.7363889	109.215111	208	6	**NO2**	11.7341111	109.182472	174	7
**AH7**	11.7358333	109.216250	236	4	**NO3**	11.7345556	109.181222	204	5
**AH8**	11.7354444	109.217264	258	7	**NO4**	11.7349583	109.180056	238	8
**AH9**	11.7349167	109.218861	260	2	**NO5**	11.7353889	109.179111	271	3
**AH10**	11.7352222	109.219500	265	2	**NO6**	11.7356667	109.177750	340	9
					**NO7**	11.7355000	109.176889	382	11
**DD1**	11.7226667	109.181306	111	3	**NO8**	11.7362778	109.175861	411	11
**DD2**	11.7193889	109.179361	158	3	**NO9**	11.7371667	109.175958	418	6
**DD3**	11.7177778	109.178333	188	4	**NO10**	11.7379861	109.175972	428	6
**DD4**	11.7165556	109.174611	243	3	**NO11**	11.7385556	109.175861	441	9
**DD5**	11.7145278	109.175083	230	2	**NO12**	11.7402639	109.175389	486	7
**DD6**	11.7150556	109.181528	212	4	**NO13**	11.7424444	109.175250	484	4
**DD1'**	11.7205833	109.182944	157	2	**NO14**	11.7456944	109.175028	474	1
**DD2'**	11.7165694	109.180903	211	2					
**DD3'**	11.7151944	109.179236	230	4	**ST1**	11.7346667	109.190583	101	4
**DD4'**	11.7152222	109.176625	224	2	**ST2**	11.7368333	109.190222	171	11
**DD5'**	11.7164167	109.177278	219	3	**ST3**	11.7391667	109.190653	251	11
					**ST4**	11.7411250	109.191597	287	1
**MN1**	11.7279830	109.189704	38	2	**ST5**	11.7436528	109.192681	326	21
**MN2**	11.7265833	109.185528	67	10	**ST6**	11.7446806	109.192500	351	18
**MN3**	11.7246806	109.182014	107	10	**ST7**	11.7461667	109.192167	378	13
**MN4**	11.7245556	109.180417	140	4	**ST8**	11.7485000	109.192833	405	15
**MN5**	11.7246944	109.179403	166	12	**ST9**	11.7511528	109.192319	392	18
**MN6**	11.7226944	109.179417	162	2	**ST10**	11.7534444	109.192875	376	21

Samples were assigned to a single representative locality point, for which geographic coordinates and elevation are given. N: number of specimens sampled in each unit. Forest paths identified with an abbreviated code (AH: Ao Ho, DD: Da Do, DH: Da Hang, MN: Mai Nha, NO: Nui Ong, and ST: Suoi Truc) and numbers as shown in Fig 1.

### Molecular methods

Each leaf beetle specimen was subject to standard DNA extraction using the DNeasy Blood and Tissue kit (Qiagen Iberia, Madrid, Spain). We used whole specimens, which were recovered after DNA extraction, mounted dry and labelled with a voucher number for future reference in the senior author's institutional collection. Individual DNA were used as template for PCR amplification of ca. 830 bp at the 3'-half of the mitochondrial cytochrome *c* oxidase subunit 1 (*cox1*) gene by using the pair of primers TL-N-3014 [37] and a modified C1-J-2183 [38]. When this primer combination failed, we amplified the same locus in two shorter, non-overlapping fragments using a suitable internal primer or its reverse complement with each of the previous ones [38]. PCR used conventional *Taq* polymerase and standard conditions with 35 temperature cycles of 30 s denaturation at 94°C, 30 s annealing at 50°C and 1 min elongation at 72°C. PCR products were purified using ammonium acetate and cold isopropanol, checked in agarose gel and sequenced in both directions using the same PCR primers and the BigDye Terminator v3.1 Cycle Sequencing kit (Applied Biosystems, Foster City CA, USA). Complementary sequences were assembled into contigs and edited using Geneious Pro 5.3.6 (Biomatters Ltd., Auckland, New Zealand) and they were unambiguously aligned manually. Sequences generated for this study have been deposited in the European Nucleotide Archive database (EMBL-EBI, Hinxton) with accession numbers LT160095-LT160588.

### Species delimitation

Species delimitation used several objective tree-based methods, whose relative performance was assessed by quantifying their match with morphospecies. Two tree-based species approaches were tested: the Generalized Mixed Yule-Coalescent (GMYC) model considering both single and multiple thresholds [39–40], and the Bayesian implementation of the Poisson Tree Processes (bPTP) model [41]. Prior to phylogenetic analyses, all data were collapsed to haplotypes and individuals sharing haplotype were reassigned to their species *a posteriori*.

GMYC was run using the "splits" package [42] for R 3.1.1 [43]. Two strategies were used to obtain ultrametric trees for GMYC analyses, including linearisation of a maximum likelihood (ML) tree obtained with RAxML 7.2.6 [44] and Bayesian inference (BI) using BEAST 1.8.1 [45]. Previous to phylogenetic analyses, the evolutionary model with best fit to the variation observed in the *cox1* data matrix was assessed based on several information criteria implemented in jModelTest 2.1.5 [46]. The selected model was a GTR+I+G in every case. The optimal ML tree was obtained with an initial step to explore the best initial rearrangement setting from a collection of 100 most parsimonious random starting trees, and a second step using these optimal settings in a multiple inference search for the best-known likelihood tree using 500 replicates. The obtained ML tree was rooted in the mid-point of the longest path between two terminals and was made ultrametric either with (1) the PATHd8 algorithm, which transforms branch lengths by local rate smoothing to accommodate deviations from a molecular clock [47]; or (2) the parametric rate smoothing method implemented in r8s 1.8 [48], whereby we obtained the optimal smoothing parameter by cross-validation using a range of values between 0.01 and 1000, and fixing the age of the root arbitrarily to 100 time units. In turn, BI was used to produce gene-trees using four Markov chain Monte Carlo chains with 50 million generations, a GTR+I+G substitution model with parameters estimated from the sample, and a coalescent tree prior, sampling trees and associated parameters every 5000 generations. A maximum clade credibility tree with relevant parameters were obtained averaging over this sample of trees after conservatively removing the initial 10% of samples using TreeAnnotator 1.6.2 [45] and Tracer 1.6 [49]. Bayesian trees were obtained both under strict and uncorrelated lognormal relaxed clocks, recording the estimated value for the parameter *ucld*.*mean*.

The bPTP method, does not require ultrametric trees and species delimitation is optimised on branch lengths, which are kept proportional to the number of mutations inferred along their path [41]. bPTP ran using the online resource "bPTP server" (http://species.h-its.org) and we used the same mid-point rooted RAxML input tree as before.

### Objective biome splitting through environmental gradient

The Núi Chúa National Park forests are not fragmented and the change in biomes and their inhabitants, from deciduous dry forest to forests typical of higher moisture environments, occurred gradually through a wide ecotone. Most of our sampling was precisely in this ecotone, but in order to quantify differences between drier and moister biomes, we explored tentatively an objective, yet artificial boundary between biomes by analysing several beetle community parameters using a sliding window approach [10]. Specifically, we measured spatial variation of intuitive parameters for community composition such as the proportion of shared species (Sørensen-Dice index; [50–51]) and species exclusivity (i.e., species exclusively found below or above the corresponding elevation threshold) between two compartments of data. We sliced our total sample in 40 m elevation intervals and made comparisons of species composition between successive adjacent intervals using incidence data based on one of our species hypothesis. (We tentatively explored smaller and bigger slices and 40 m seemed a good compromise between number of sliding steps to show trends in beta-diversity changes, and amount of beetle species diversity covered in each analysed interval.) This procedure assumes that the study site overlaps an area where two communities meet diffusely, one adapted to drier environments at lower elevations and reaching as high as these adaptations allow, and one adapted to moister environments at higher elevations and reaching as low as possible. This overlap may imply an area of increased diversity in the ecotone, harbouring species from both communities, with relative drops in diversity at the "hard" edges, far from the centre of the respective domains, where environment and competition impose harder restrictions to the expansion of each community.

### Species richness estimation

Our leaf beetle survey in Núi Chúa is the first in this National Park and there is no reference catalogue for the total expected diversity of Chrysomelidae in this region of southern Vietnam. In order to assess, even if in exploratory terms, our degree of success in sampling local diversity, we applied nonparametric and rarefaction methods (factor 3x) based on incidence data to investigate expected species richness in different geographic (path) and ecological (biome) partitions of the data. Species accumulation curves were obtained based on the results of different species delimitation approaches and a range of species richness estimators calculated using 100 sample order randomisations in EstimateS 9.1 [52]. Chao2 indexes [53] were estimated with bias correction, except when the estimated coefficient of variation for abundance and/or incidence distributions was above a certain threshold (CV>0.5), and the classic Chao2 index was used instead [52].

### Analysis of diversity patterns

Sørensen's and Jaccard's indices were calculated as implemented in EstimateS 9.1 [52] in order to compare species diversity between forest paths following [54]. The analysis of changes in community composition focused on differences in species composition among data partitioned according to samples (i.e., forest paths), biomes and the intersection of both aspects of data. In every case, we described the components of dissimilarity between sites according to a decomposition of differences due to species replacement (turnover, measured as β_SIM_) and species impoverishment (nestedness, measured as β_SNE_) as proposed by [55]. There are a number of strategies that have been proposed to partition total beta-diversity in these components, including two competing frameworks splitting beta-diversity in turnover and nestedness, as proposed by [55], or in species replacement (β_repl_) and species loss or gain (i.e., richness; β_rich_), as suggested by [56]. Particularly the latter index has been suggested to outperform the supposedly analogous measure of nestedness, which may underestimate species loss and gain, and these indexes would be more robust to undersampling, minimising biases in beta-diversity estimation when dealing with incomplete species inventories [56–57]. However, Baselga and Leprieur [6] have shown recently that these alternative decompositions actually represent different concepts and therefore different underlying processes generating the observed patterns, also in the interpretation of species replacement, with β_SOR_ but not β_repl_ faithfully reflecting species turnover independently of species richness differences. Based on the latter considerations, here we adopted the framework proposed in [55] and the corresponding dissimilarity measures were calculated using the package "betapart" [58] for R 3.1.1 [43]. However, to facilitate future contrasts of these measures and in order to reveal commonalities that could reflect the same structuring processes, the alternative indexes were also calculated using the package "BAT" [59] for R 3.1.1.

## Results

### Observed species diversity in Núi Chúa

DNA was extracted from 520 leaf beetle specimens, but only 494 produced *cox1* sequences (344 haplotypes) for objective species delimitation. These samples were classified as belonging to 140 morphospecies, based on the study of external morphology and male and/or female genitalia when there existed doubts (e.g., colour polymorphism). Most specimens belonged to the Chrysomelidae subfamilies dominant in the tropics, namely Eumolpinae (221 specimens, 41 morphospecies) and the assemblage of galerucines (146 specimens, 42 morphospecies) and flea beetles (49 specimens, 23 morphospecies) (Table 2). Other subfamilies were less frequent and, with the exception of Cryptocephalinae and Hispinae (including tortoise and hispine beetles), were missing in some paths. Morphospecies diversity per path ranged between 22 and 58, with transects reaching lower elevations (AH and DD) showing lower species counts. Despite less intense sampling at higher elevations, species diversity above and below 300 m was similar, 85–95 morphospecies, respectively (Table 2).

**Table 2:** Chrysomelidae diversity in the Núi Chúa National Park.

			Sample						Biome	
	N	Species	AH	DD	MN	NO	ST	DH	<300m	>300m
Alticinae	49	23/25	4/4	5/5	6/6	8/8	6/7	6/6	17/18	12/12
Bruchinae	7	3/3	-	-	2/2	2/2	-	-	3/3	-
Chlamysinae	3	2/2	-	-	-	1/1	1/1	-	-	2/2
Chrysomelinae	2	2/2	1/1	-	-	-	-	1/1	1/1	1/1
Clytrinae	12	7/7	1/1	-	3/3	-	4/4	3/3	5/5	4/4
Criocerinae	2	2/2	-	-	-	-	2/2	-	-	2/2
Cryptocephalinae	14	9/9	4/4	1/1	1/1	1/1	5/5	1/1	7/7	4/4
Eumolpinae	221	41/51	12/13	7/8	20/22	18/21	19/20	18/19	26/32	30/33
Galerucinae	146	42/45	8/8	6/6	17/17	16/17	19/20	9/9	28/29	27/29
Hispinae	38	9/9	1/1	3/3	8/8	2/2	2/2	1/1	8/8	3/3
Total	494	140/155	31/32	22/23	57/59	48/52	58/61	39/40	95/103	85/90

For each subfamily, both the number of specimens (N) and species are given, the latter based on morphospecies and bPTP delimitation, respectively. Data are shown for the total assemblage, per forest path, and for two elevation ranges dominated by dry and moister forest biomes.

**Fig 3:**
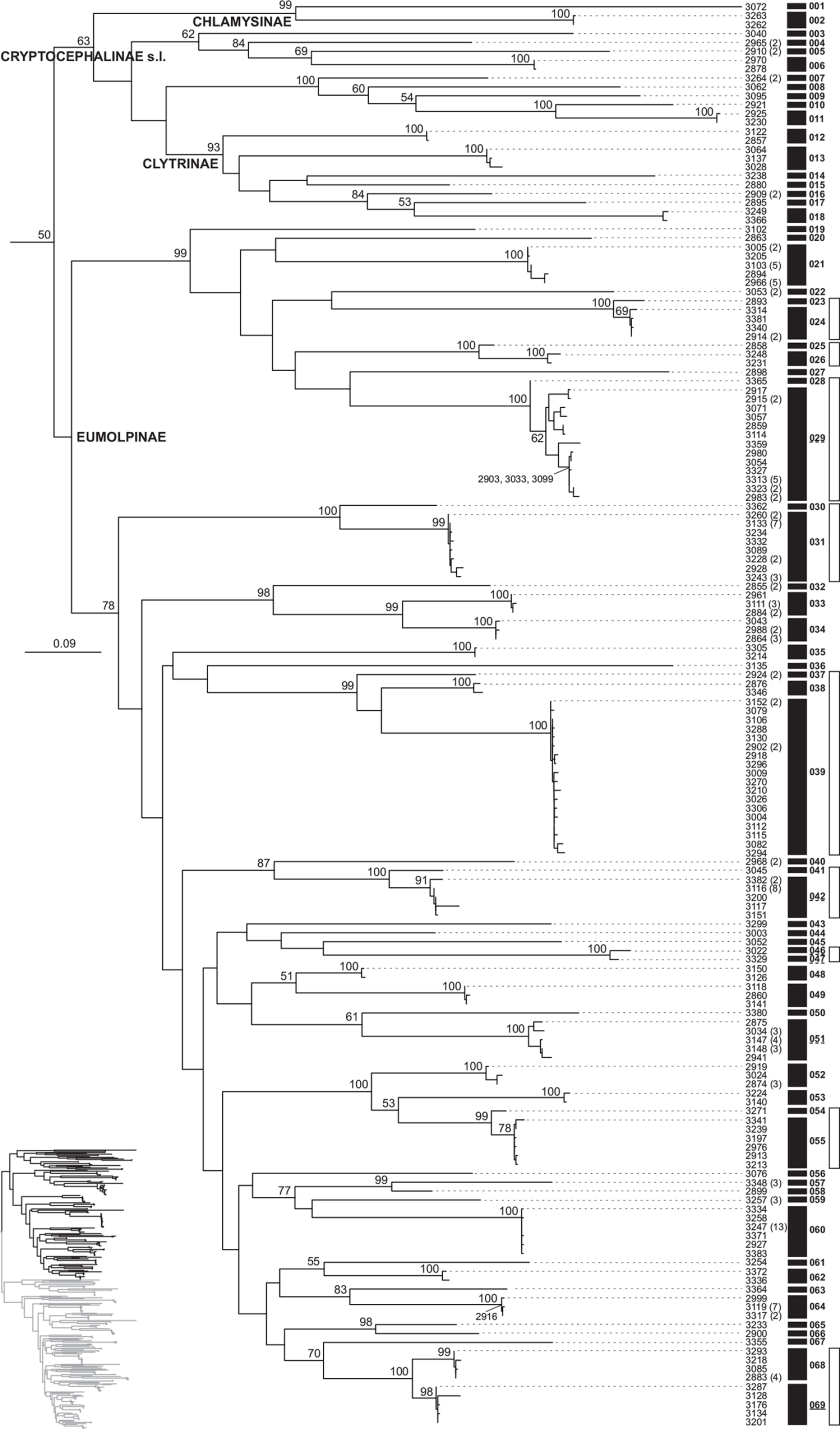
MtDNA tree-based species delimitation of Chrysomelidae (species 001–069) in the Núi Chúa National Park. Clade of the Maximum Likelihood *cox1* haplotype tree of Chrysomelidae from the Núi Chúa National Park including the Cryptocephalinae and Eumolpinae (the sister clade is shown in Fig 4). Tip numbers are individual haplotypes matching the voucher number of the source specimen; when several specimens shared a haplotype, their total number is indicated. Numbers at nodes represent bootstrap support. Numbered black boxes are units based on the bPTP species delimitation method and white boxes morphospecies showing disagreements with bPTP species.

**Fig 4:**
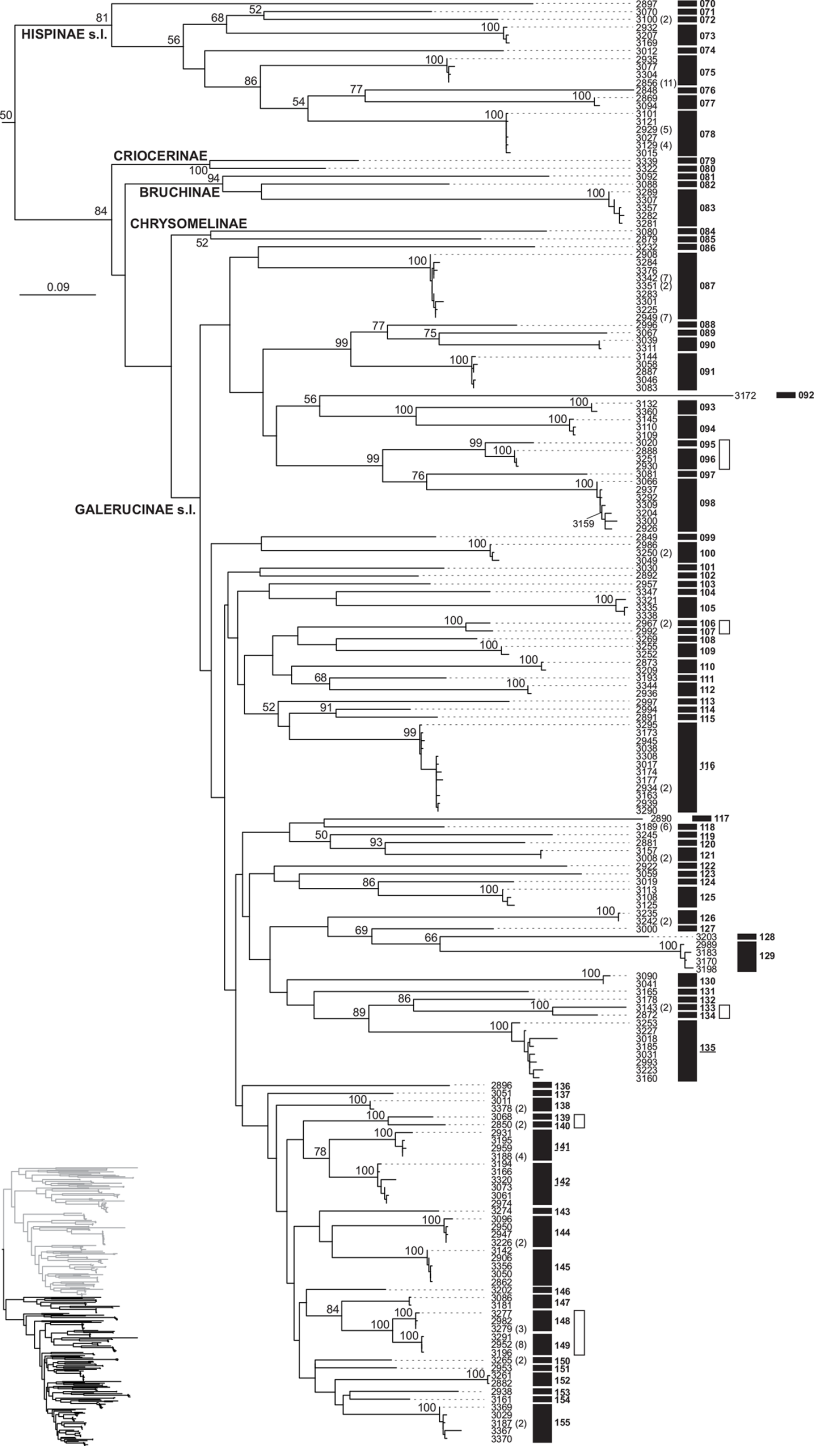
MtDNA tree-based species delimitation of Chrysomelidae (species 070–155) in the Núi Chúa National Park. Clade of the Maximum Likelihood *cox1* haplotype tree of Chrysomelidae from the Núi Chúa National Park including the Hispinae, Criocerinae, Bruchinae, Chrysomelinae, Galerucinae and Alticinae (the sister clade is shown in Fig 3). Tip numbers are individual haplotypes matching the voucher number of the source specimen; when several specimens shared a haplotype, their total number is indicated. Numbers at nodes represent bootstrap support. Numbered black boxes are units based on the bPTP species delimitation method and white boxes morphospecies showing disagreements with bPTP species.

Tree-based species delimitation yielded species estimates ranging between 155 (bPTP from ML tree) and 186 (GMYC with multiple threshold on an ML tree linearised using r8s). With the exception of GMYC with multiple thresholds, all methods produced remarkably similar species hypotheses among each other and compared with the preliminary separation of morphospecies (Table 3). The highest match with morphospecies was obtained with bPTP, whereby 90% of the morphospecies were recovered using this methodology (Figs 3 and 4). The highest reliability of this method for our dataset possibly resides in its independence from clock-like behaviour of data, which in our case showed a slight deviation from the molecular clock (moderate variation in substitution rates among branches: *ucld*.*stdev* = 0.4092±0.0033). However, both single-threshold GMYC attempts at an ML tree linearised with PATHd8 and the clocklike Bayesian tree performed nearly as well, where the match reached 88.6% in both cases (Table 3). Accordingly, tree-based methods, again with the exception of those using multiple-threshold GMYC species assessment, showed good agreement with each other. Thus, only eight bPTP species showed further splitting with one or most other single-threshold methods, and two were merged into one unit (in agreement with morphospecies assessment). All disagreements between morphospecies and the bPTP approach represented the split of the former into two or, in one case, three units (Table 4). A relatively high proportion of species inferred from haplotypes were singletons: 60% for bPTP assessment and 58.6–64.6% for GMYC tests. When individuals were reinstated based on haplotype data, the amount of singletons, species represented by a single individual in our dataset, dropped to 49.7% in the most conservative bPTP case. Fourteen morphospecies were further split by bPTP and other methods, except molecular species 046 and 047, which were retained as a single unit by most GMYC tests. These phylogenetic splits generally separated a single most divergent haplotype in a monophyletic assemblage (average p-distance: 0.096 ± 0.080), and these rarely represented cases of allopatric samples in terms of their source transect or their biome allocation (Table 4).

**Table 3:** DNA-based species delimitation of Chrysomelidae in the Núi Chúa National Park.

Tree	Linear.	Thresh.	Entities [CI]	Clusters [CI]	L_GMYC_	Agree	Split	Merge
ML	r8s	S	178 [175–181]	63 [62–65]	500.063	122	18	0
		M	186 [183–186]	62 [61–62]	505.887	119	21	0
	Pd8	S	160 [155–163]	65 [64–66]	522.670	124	16	0
		M	161 [152–166]	76 [73–76]	527.336	39	20	81
BI	SC	S	162 [157–166]	67 [65–67]	2480.504	124	16	0
		M	165 [153–165]	94 [91–96]	2491.387	39	21	80
	ULN	S	164 [158–166]	66 [66–68]	2463.070	122	18	0
		M	173 [170–174]	67 [66–67]	2466.495	119	21	0
ML	-	bPTP	155	-	-	126	14	0

Species delimitation used *cox1* sequence data and alternative tree optimisation (ML: Maximum Likelihood; BI: Bayesian Inference) and tree linearisation (see main text) methods, as well as single (S) or multiple (M) thresholds for GMYC species delimitation. The number of species and clusters of more than one sequence with their respective confidence intervals are given for GMYC results, as well as the likelihood of the fit of the model. The fit of DNA-based entities with morphospecies is given as the absolute number of perfect matches, and morphospecies split or merged by each method.

**Table 4:** Disagreements of bPTP species delimitation and morphospecies of Chrysomelidae in the Núi Chú National Park.

bPTP	No. ind.	No. loc.	Same path?	Same Biome?	mean d ± SD	Reassessment morphology
023–024	(1,5)	5	Yes	Yes	0.058 ± 0.005	Colour differences
025–026	(1,2)	3	No	Yes	0.043 ± 0.006	No differences
028–029	(1,23)	18	Yes	Yes	0.125 ± 0.005	No differences
030–031	(1,18)	17	Yes	Yes	0.046 ± 0.004	Different size and vestiture
037–039	(2,2,20)	19	Yes	Yes	0.304 ± 0.007	No differences
041–042	(1,13)	12	Yes	Yes	0.228 ± 0.006	No differences
046–047	(1,1)	2	No	No	0.019 ± 0.005	No differences
054–055	(1,6)	7	Yes	Yes	0.059 ± 0.004	No differences
068–069	(7,5)	12	Yes	Yes	0.070 ± 0.006	No differences
095–096	(1,3)	4	Yes	Yes	0.107 ± 0.007	No differences
106–107	(2,1)	3	No	No	0.031 ± 0.006	No differences
133–134	(2,1)	3	No	Yes	0.071 ± 0.008	Colour differences on head
139–140	(1,2)	3	No	No	0.109 ± 0.011	Different species (penis)
148–149	(5,10)	13	Yes	Yes	0.067 ± 0.005	No differences

Species identification is provided with bPTP species numbers as in Figs 3 and 4, giving number of individuals, number of involved localities, and information on spatial segregation according to forest path and biome. Mean genetic divergences between bPTP lineages splitting a morphospecies are shown, along with a reassessment of morphospecies.

### Objective delimitation of ecotone

**Fig 5:**
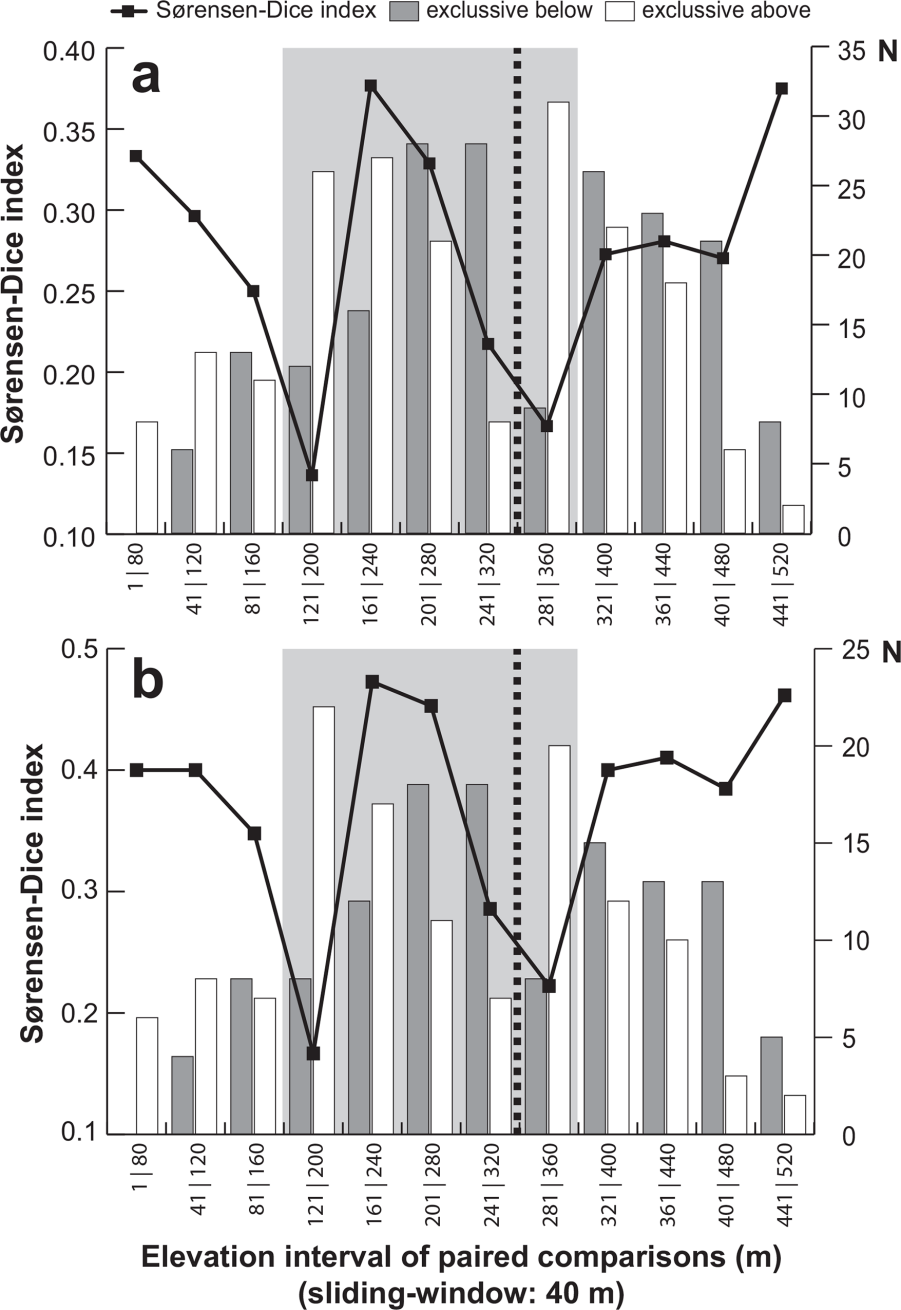
Patterns of shared leaf beetle species and species exclusivity along an elevation gradient in the Núi Chúa National Park. Species distribution patterns are shown including (a) and excluding (b) singletons. Each data point compares adjacent elevation segments of 40 m. The middle gray area identifies a middle increase in shared diversity and the thick discontinuous line a shift in species exclusivity tentatively placed at 300 m a.s.l.

Based on our best objective species delimitation hypothesis (bPTP method), the sliding window approach yielded relatively stable values (0.25–0.37) for the Sørensen-Dice index of species homogeneity in most comparisons, dropping below 0.16 in the comparisons side-to-side of 160 m and 320 m, respectively (Fig 5A). We propose this elevation segment as the transition area between dry and moister biomes. Moreover, within this broad area representing a gradual shift in the composition of communities, we also detected a change in the segment comprised between 240 m and 360 m in the trend of species exclusivity considering the lower or upper slices of each comparison. This area, specifically the midpoint at 300 m, was considered the strongest boundary of the ecotone for further comparisons. The same pattern and trends were obtained after excluding singletons, logically with slightly increased compositional similarity and reduced species exclusivity counts (Fig 5B).

### Expected diversity in Núi Chúa

Table 5 shows different incidence-based nonparametric estimates of species richness using the available samples and different partitions of data. Path DH, sampled only once and rather unevenly, despite its observed high species diversity, produced the least consistent estimates of species richness, and was not further considered. Paths AH and ST appeared as these better sampled, with total species richness estimates generally doubling at most sampled diversity. Forest paths DD, MN and NO were less well sampled, with species richness more than twice (except for Jack1) and up to 4.2x (Chao2 estimate of DD) higher than the number of species sampled; these estimates also showed proportionally higher variances. These partitions of data offer a tentative exploration of sampling success, but most reliable estimates can be obtained considering the regional sample which globally represented about 51–68% (depending on the estimator) of the expected diversity in the area of study. Data partitioned according to elevation showed that both the drier (< 300 m) and moister (> 300 m) parts of the environmental and ecological gradient similarly achieved about 50–66% of their expected diversity, although sampling above 300 m proved less efficient.

**Table 5:** Incidence-based species richness estimators of Chrysomelidae in the Núi Chúa National Park.

Data	N	Rarefaction (S)	ICE	Chao2	Jack1	Jack2
AH	27^a^	48.7±10.09	72.00	51.4±14.94	45.0±4.44	56.73
DD	23	51.2±11.72	76.48	96.6±63.16^b^	39.4±3.63	52.61
DH	40	99.9±16.98	329.83	173.2±73.69	69.6±5.60	90.45
MN	57^a^	120.4±17.61	170.85	213.9±86.49^b^	95.3±7.90	126.77
NO	52	102.1±15.64	146.52	142.8±46.47^b^	86.4±6.33	112.53
ST	58^a^	101.8±14.37	150.84	120.2±28.48^b^	93.1±6.63	116.47
<300m^c^	94	147.4±15.18	201.59	156.2±22.29	141.2±14.44	167.15
>300m^c^	74	121.5±13.99	220.64	129.0±20.92	110.7±13.87	126.16
Total^c^	133^a^	197.3±16.31	260.18	205.8±22.44	195.4±13.30	227.80
Total^d^	155	226.3±17.08	301.09	234.5±22.58	230.0±12.24	268.13
Total^e^	151^a^	225.3±18.43	280.94	241.8±27.62^b^	227.8±12.40	271.91

All species richness estimates (and their standard deviation when appropriate) are based on the results of bPTP species delimitation (N).

^a^ A few samples could not be assigned to a specific point locality.

^b^ Computed with classic estimator [52].

^c^ Estimates excluding the poorly sampled DH forest path.

^d^ Sample split by forest path.

^e^ Sample split by collection points.

### Diversity patterns in Núi Chúa

Compositional similarity between data partitions (excluding DH, with much lower similarity with other paths, and standing out as a sampling outlier based on previous results) showed values between 0.29 and 0.46 based on Sørensen's and 0.14–0.30 based on Jaccard's indexes (Table 6). Global compositional difference between biomes was in the same range, with β = 0.42 (Sørensen) or 0.27 (Jaccard).

**Table 6:** Chrysomelidae species composition similarity among forest paths in the Núi Chúa National Park.

	Ao Ho	Da Do	Mai Nha	Nui Ong	Suoi Truc
**Ao Ho**	-	0.170	0.197	0.183	0.192
**Da Do**	0.290	-	0.171	0.136	0.135
**Mai Nha**	0.329	0.292	-	0.233	0.224
**Nui Ong**	0.309	0.240	0.378	-	0.298
**Suoi Truc**	0.322	0.238	0.366	0.460	-

The dissimilarity measures are based on Sørensen (below diagonal) and Jaccard (above diagonal) indexes.

**Fig 6:**
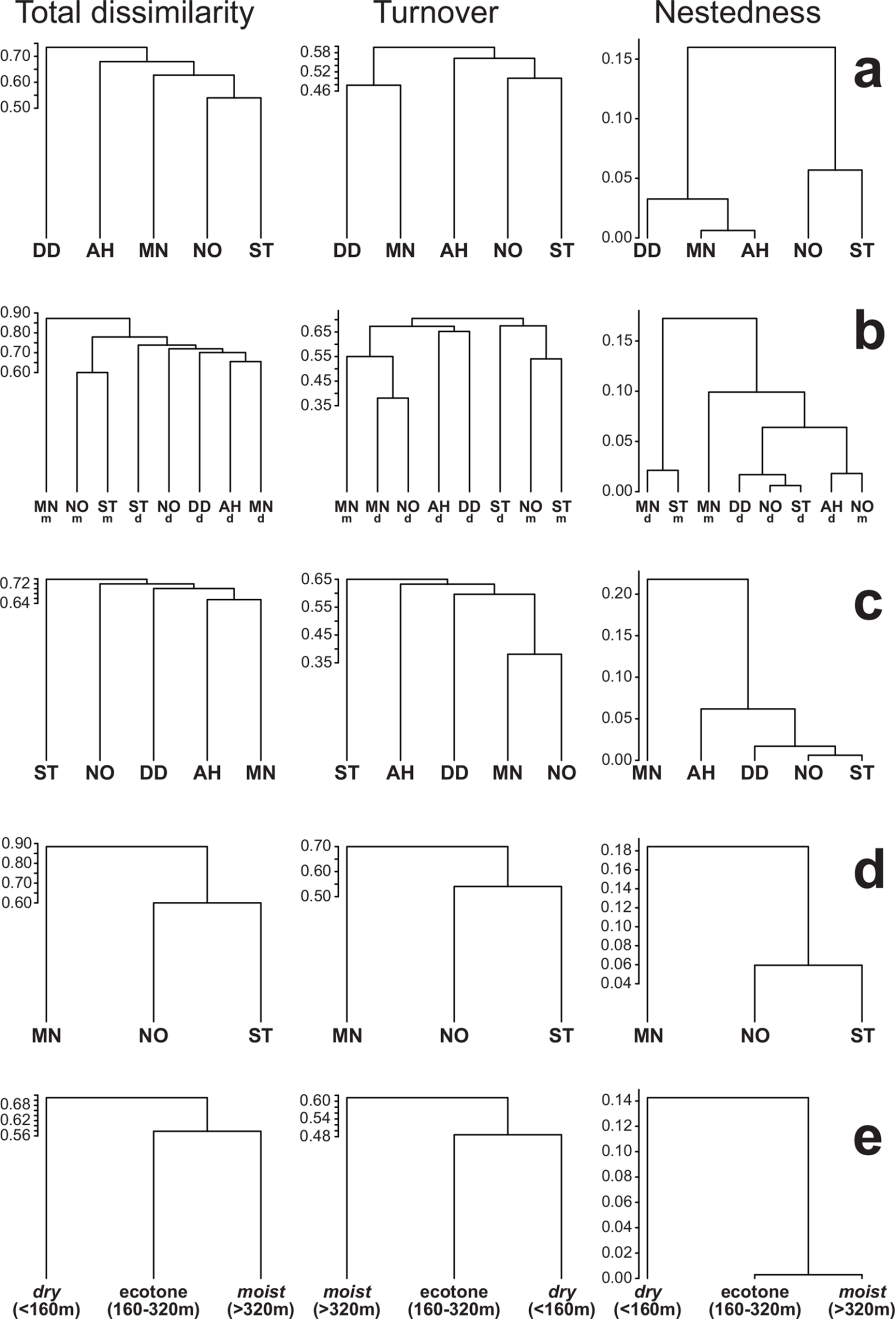
Beta-diversity patterns of leaf beetle communities in Núi Chúa National Park and their decomposition in turnover and nestedness components. Clustering of (a) transects, (b) biomes within forest paths, (c) lower/drier range of sampled area, (d) higher/moister range of sampled area, and (e) altitudinal discrimination of biomes in Núi Chúa based on leaf beetle species dissimilarity and their turnover and nestedness components of beta-diversity measured using the framework proposed by [55].

Nui Ong and Suoi Truc, transects sampled to higher elevations, showed the highest similarity, and differences among forest paths were mostly due to species replacement in most cases, except in the case of the aforementioned paths where there was a 10% contribution of dissimilarity due to nestedness (Fig 6A). Even though differences were small, the communities from the drier part of the sampled area (below 300 m) clustered together, with uneven contributions from species turnover and nestedness components of dissimilarity, although the former was always higher (Fig 6B). The respective communities in the common, lower part of forest paths showed rather homogeneous similarity (~28–34%). Differences were mainly due again to species replacement, except in the case of MN, second to highest in species richness despite reaching relatively low elevation (364 m), which showed a high nestedness component of total dissimilarity (Fig 6C). A similar pattern was obtained when only the communities in the higher and moister areas were analysed, reflecting a higher similarity of paths NO and ST, the main contribution to overall highest similarity of these two paths (Fig 6D). Using the tentative delimitation of the ecotone outlined above as the guide to partition the communities in our total sample, the community in this intermediate transition area showed highest overall similarity with that in the higher, moister forest. However, species turnover was lower with the communities in the typically sclerophyll forest (~48 vs. 61%), an effect that was in great part modulated by a relatively high dissimilarity due to nestedness (~14%) of communities in higher elevations relative to these in the driest and lowest area of our study site (Fig 6E). Beta-diversity partitioning using βrepl and βrich produced similar clustering, but with a considerably higher contribution of the richness component as advised by [56] (S1 Fig).

## Discussion

### Overwhelming species-richness of Chrysomelidae in Núi Chúa forests

The study of hyperdiverse insect groups always represents a challenge for the analysis of community composition in tropical ecosystems, even at small, very local scales [60]. Besides reduced chances to sample assemblages comprehensively, these groups generally lack updated taxonomic revisions or species catalogues, many species remain unrecognised and awaiting description, and there is no available expertise to produce informed species sorting (let alone naming of species). Thus, many studies characterising tropical insect faunas typically resort to parataxonomic practice and morphospecies bins as proxies to species diversity (e.g., [61]). However, this shortcoming for community analyses can be easily circumvented employing objective DNA-based delimitation methods [16]. Moreover, these approaches are particularly well suited for studies of local assemblages. At reduced geographical scales, species delimitation using fast-evolving standard DNA markers yields very accurate results [62]. Local samples generally represent a fraction of the total genetic variation of the species and there is a trend towards increased interspecific genetic divergences with sympatric closest relatives [62–63]. An additional strength of DNA-based methods for community ecology is their potential to retrieve cryptic diversity [64], which, if unnoticed, obviously undermines the perception of diversity [65]. Here, we combined a careful rendition of morphospecies with alternative methodologies for DNA-based species delimitation, consistent with 155 species present in our local leaf beetle sample in Núi Chúa.

The next concern affects our perception on how representative is our sample of the chrysomelid communities in the surveyed area. We are aware of sampling limitations possibly biasing our species richness estimates (e.g. [5]), as identified by the use of theoretical approaches to total species richness, but also by unbalances in the representation of certain taxonomic groups (Table 2). Thus, species richness estimators suggest that in the less favourable case we would have succeeded at sampling 51.5% of total diversity in the prospected environments or, alternatively, above 68% in the most favourable case. This is in the same range as achieved in similar studies of tropical leaf beetle communities, even those using more varied or systematic collection techniques (e.g., [66–69]).

With 155 leaf beetle species and species richness estimates in the order of 225–300 species in the prospected area, and in part influenced by a relatively small grain and extent of the study [5], we perceive that local alpha-diversity in Núi Chúa is very high. This subjective perception of diversity can be modulated by reference to other systems and scales. Examples of leaf beetle species richness at small regional or local scales in tropical forests could be the 200 species in an 11.6 km long transect covering an elevation extent of 760 m in Peregrina Canyon (Tamaulipas, Mexico; [68]); above 400 species estimated in an elevation gradient between 1000 and 3000 m a.s.l. in mountain forests of Ecuador in an area of ~140 km^2^ [69]; or 510 species of canopy (from 10–40 m of elevation) leaf beetles in one dry and one wet evergreen forest in Panama located more than 55 km apart [70]. Even compared to the latter estimates, ours stands out as relatively high, again considering the reduced scale, extent and subtle ecological gradient considered here.

Indeed, our results must necessarily offer a conservative view to total species richness in Núi Chúa, since collecting efforts were very much restricted by logistics, sampling difficulties in occasionally very dense forest, characteristically low beetle densities, and collecting restricted to the lowest vegetation stratum in the forest. Charles and Basset [67] evaluated differences in the communities of leaf beetles between the canopy and understorey both in dry and wet tropical forests in Panama. They found similar species numbers between forest types, but a 1.5–3 fold increase in species richness in canopy communities relative to these in lower plant strata, whereby only one fourth of the species were shared between communities. If a similar diversity pattern would occur in our system, applying these factors, we could expect total leaf beetle diversity in the studied area to be well above 500 species and maybe approaching twice this figure.

### Leaf beetle species diversity is not distributed homogeneously

The proportion of shared leaf beetle species between forest paths and types in Núi Chúa was relatively low (Table 6), thus producing high dissimilarity values, typically above 0.60, and a dominance of species turnover (factor = 0.41–0.99) over nestedness (and species richness contributions, in the framework of [56]) in a short elevation and environmental gradient (Fig 6). These values are similar to those obtained, e.g., for scarab communities sampled along a 300 km transect in Costa Rica (Sørensen index of compositional similarity = 0.21–0.46; [71]). Considering the relatively small scale of the study, and similarly to our assessment of alpha-diversity, our perception is that there is a high contribution of beta-diversity to whole assemblage diversity.

We have not sampled total leaf beetle diversity in the studied area, and this can potentially bias our understanding of beta-diversity as well, but it is difficult to predict in which direction. Thus, beta-diversity may be overestimated because our sampling represents an incomplete catalogue of species actually present in any given sampling point, but it could be underestimated too because of a failure to sample rare species in each locality [16]. Ideally, these opposing effects resulted in beta-diversity trends accurate enough, as would be recovered with a more comprehensive sample. Sample-unit size may have an important effect also on the perception of beta-diversity [11, 72]. Small sample-units relative to total sampled area may increase dissimilarity due to variability in species occupancy; conversely, large sample-units may have the opposite effect by including species removed from their optimal environment. Our sample-unit size is small, and this could introduce some biases too, inflating beta-diversity in local sampling [10, 14]. However, considering that the total area sampled is also local and that sampling effort was repeated through time, every time in slightly different spots and plants, it seems less likely that the sample-unit bias is conditioning heavily our results. Moreover, if sample-unit size induces no bias, beta-diversity should be inversely correlated with distance (distance-decay pattern) or, at least, not much higher for shorter distances neither between nor within paths. Albeit with very slight distance decay for composition similarity, this seems to be the recovered pattern for our data (S2 Fig). Taking into account that we tried to cover the area where changes in patterns were expected (the ecotone) and that this pattern emerged, the potential negative impact, if any, of these sampling limitations may not be so worrisome [5].

Highest global dissimilarities were obtained after a combined effect of spatial segregation but also ecological diversity. Thus, samples at lower elevations, exclusively within dry forest, and more distant (AH and DD), contributed higher beta-diversity to the whole assemblage (Table 6, Fig 6A). The highest beta-diversity (with total dissimilarity ranging ~0.60–0.90) results from partitioning the data precisely based on these two spatial criteria, namely paths and elevation (biome), whereby samples collected in the drier biome tend to cluster together, if with relatively high total dissimilarity (~0.74). We identified very similar species numbers between lowland and higher elevations (89 *vs*. 74 species, respectively, below and above 300 m), only slightly lower in the latter despite lower sampling effort, counted as number of sample-units (42 *vs*. 18, respectively). The proportion of singletons at both elevations was very similar (38.2% and 35.1%, respectively), suggesting that rare species and/or intensity of sampling were relatively well balanced. These data denote higher species richness at higher elevations and these leaf beetle communities also show higher beta-diversity and somewhat more species turnover compared to lowland communities (Fig 6C and 6D). This type of pattern diversity (*sensu* [73]), higher species diversity and higher or similar beta-diversity at higher elevations, is not typical of tropical elevation gradients (e.g., [28]). Instead, alternative scenarios have been reported, including the reversed pattern in most cases (e.g., [12, 15, 23, 74]) or pattern homogeneity (e.g., [21, 26]). This heterogeneity of pattern diversities suggests their strong dependence on taxonomic, ecological and historical factors, but also on scale and the extent of the gradients, but at present we cannot discard sampling biases either [5, 11–12].

### Potential causes of small-scale heterogeneity of leaf beetle assemblages

Documenting differences in the structure of communities is relevant, but unravelling the causes of diversity patterns is one of the most important goals of community ecology, among other reasons because it can help addressing specific conservation concerns and practices. Considering the small scale of our study, marked differences in community structure with a significant contribution of species turnover may be indicative of a relatively high proportion of specialists, i.e. admitting that there is a direct correlation between host specificity and beta-diversity when comparing sites along an ecological gradient. The nestedness and richness components of beta-diversity could reflect community changes associated to species loss due to habitat deterioration (considering diversity gradients from the higher-elevation forest to cultivated lowland) or species gain due to increase in habitat complexity (looking at the gradient in the opposite direction). Unfortunately, in our first approach to the diversity of leaf beetles in Núi Chúa forests, lack of data on other factors—biotic or abiotic and, critically, at a similar scale—precludes specific testing of assembly hypotheses. Thus, we can only conjecture on the causes of leaf beetle assemblage heterogeneity as an exercise to generate testable hypotheses on this system.

Elevation is a surrogate for several intercorrelated environmental gradients, but which one or combination of several may be conditioning the assembly of communities is controversial [5]. Among potential factors, temperature is one of the most influential [75]. Temperature, but also other determinants, supposedly has differential effects on the species assembling the communities, either directly through their physiological tolerance or indirectly through effects on other species [8, 76]. Temperature directly affects photosynthesis, metabolism and nutrient supply from the soil, and thus filters plant species according to functional traits and specific adaptations, controlling the plant community [8]. In turn, temperature combined with moisture (although moisture is not an elevation phenomenon; [75]) are recognised as strong drivers of animal community composition [8, 15, 23, 76–77]. As for indirect effects, and of particular interest in the case of herbivorous insects with different types and degrees of specialisation, the availability of plants that they can use as food necessarily conditions their distribution too [76].

We hypothesise that it is precisely a combination of direct and indirect effects of environmental gradients, the latter conditioning the structure of plant communities along the forest transition, that modulate small-scale chrysomelid diversity in Núi Chúa. First, environmental control has been recognised as the dominant structuring process at local scales, with species able to display narrow resource use specialisation and tracking of environmental gradients at very fine spatial scales, while spatial effects may be more important at larger scales [10, 14]. Second, even if it is still contentious how these drivers of beta-diversity interact with scale, it is generally assumed that insect communities, particularly these of herbivore insects, change rapidly at least with marked elevation gradients [16]. This diversity pattern is generally explained by plant species turnover, so that host-plant availability is the main factor conditioning species ranges [16, 28]. Nonetheless, we consider this factor of lower relative importance as driver of community structuring, or certainly a hypothesis to test in this system. Host-plant restrictions to occupancy may be true for individual species and these highly specialised. However, leaf beetle communities represent different degrees of specialisation, as well as a variety of host-plant associations for the different species involved. Therefore, when species turnover (diversity component useful to recognise processes shaping community assemblage; [11]) is realised at the community level, it suggests that the conditioning factor is not as much the host-plant *per se* as the environmental determinants of the plant community, very much in agreement with the viewpoints of Novotny et al. [21]. Moreover, while plant species abundance can show strong regional variation in tropical forests, herbivore insect specialisation is typically realised at higher plant taxonomic levels, thus host plant patchiness imposes fewer restrictions for their dispersal [16].

Host-plant associations and the degree of specialisation of herbivorous insects interact with plant community structure as contemporary ecological processes explaining at least part of herbivore assemblages, also at reduced scales. However, these factors underlie historical explanations as well, related to when and where the specific adaptations evolved. But historical and spatial explanations of beta-diversity typically consider other processes, such as vicariant speciation and isolation/dispersal. These explanations for diversity patterns, as opposed to these in the domain of contemporary ecological processes, are generally invoked at large scales [5]. Indeed, part of the controversy on the importance of niche- versus dispersal-based community assembly may be grounded on the different scale used in the particular studies [11]. Isolation also becomes important in regional scenarios of fragmentation, whereby alpha-diversity declines and beta-diversity responds depending on loss of rare and/or dispersal of opportunistic species (e.g., [11, 78–79]). Here, we analyse a reduced local scale and through a relatively healthy and subtle ecological transition without habitat fragmentation (except some effects at lower elevations due to subsistence agriculture) or environmental heterogeneity [14]. Therefore, dispersal is not considered *a priori* an important force shaping leaf beetle local community structure.

In summary, considering the small geographic scale and gradual ecological change in our system, we explain high beta-diversity in terms of environmental, microclimatic conditioning (directly on insect physiology and indirectly on plant community), but it would be interesting to investigate the role of biological interactions (mostly competition) and ecological specialisation in enhancing this diversity. One approach would analyse spatial or niche segregation of sister o closely related species pairs. We currently have negligible data on species pairs to test these structuring hypotheses, as we still miss a large proportion of species and only have a preliminary idea about their ranges. However, focusing on these species representing disagreements between morphology and DNA-based diagnosis, which could denote cryptic species and, in any case, very close genetically, we have very few convincing instances of this type of segregation (Table 4). Information on host-plant specialisation will be critical to understand these processes too.

### Preserving the small and the intangible in Núi Chúa

Previously a Nature Reserve, Núi Chúa became a National Park, the highest protection figure established by the Vietnamese administration, slightly over a decade ago. The park encompasses some 24,300 ha, including over 16,000 ha of strict forest protection. Precisely, one of the main assets of the National Park is the relatively good condition and largely intact biodiversity of its montane forests, but also this preserved in the restored lowland forests that include one of the World's biomes of highest conservation priority, the Southern Vietnam Lowland Dry Forest (status: Critical/Endangered; World Wildlife Fund, Washington D.C.). This type of tropical dry broadleaf forest once occupied a larger extent in coastal lowlands of this extremely dry and hot region of southern Vietnam, rainshadowed by the southern Annamite Range. At present it is estimated to withstand only 10% of its original range, and in any case heavily fragmented and disturbed, suffering a burgeoning pressure from human activities [36]. Núi Chúa National Park remains therefore as one of the last enclaves and opportunities to protect the high and emblematic diversity of tropical dry forests in this part of the world which, despite of its strict protection and involvement of local communities in conservation, faces a gloomy perspective in face of its isolation.

Our primary motivation to investigate chrysomelid diversity and potential turnover in the ecological succession from dry lowland into moister forests in Núi Chúa was the urgency to enhance the knowledge on the biodiversity of the National Park to boost and reinforce conservation initiatives. We also intended to enrich protection concerns and initiatives beyond charismatic organisms, such as plants, mammals, reptiles or birds, already recognised as values of the Park. In the tropics, hyperdiverse insect groups can contribute enormous power and finesse to community ecology and conservation biology, and at the same time are among the great losers, disappearing from altered biomes in many cases without leaving a trace of having existed ever [80]. The study of these animal groups (the 'small') has been historically hampered by taxonomic drawbacks, but neglecting them for community studies and conservation programmes is not tenable anymore, thanks to the simplicity of large-scale DNA-based biodiversity assessment (e.g., [39, 81]).

Our most relevant finding and with clear implications for conservation are the high local chrysomelid diversity and, especially, the relatively high and similar turnover among samples and at different elevations. There is an important contribution of beta-diversity to overall regional diversity, and effective protection measures in Núi Chúa should take into account this high diversity heterogeneity [11]. Knowing how beta-diversity correlates with spatial scale is important to understand risks of extinction and derive efficient conservation policies. Given the small-scale extent of our study and extrapolating from the strongly zoned diversity in this area, expanding conservation areas or specific safeguard measures should incorporate even higher diversity benefiting from protection. The downside is that local habitat disturbance can have dramatic effects on survival chances for the populations or downright the species in the case of narrowly endemic taxa [15]. Moreover, we purposely focused here on a reduced scale, which emphasises the role of environmental determinants of diversity. However, since the processes responsible for community composition may be different at different scales (and for different taxa with different life-histories), multiscale sampling approaches should further improve the success of any conservation initiative trying to preserve all relevant processes (the 'intangible') for maintenance of diversity [2, 5, 11]. Thus, besides examining increased spatial extents, it would be important to study leaf beetle community dynamics through time to evaluate the permeability of the ecotone to environmental fluctuations as well as the width of species' niches and their biotic interactions, also through time. Together with predictions based on scenarios of climatic change, this temporal and adaptive view would help predicting the elasticity of the system to cope and adapt to change [15, 82].

## Supporting Information

S1 Fig Beta-diversity patterns of leaf beetle communities in Núi Chúa National Park and their decomposition in species replacement and gain/loss (= richness) components. Clustering of (a) transects, (b) biomes within forest paths, (c) lower/drier range of sampled area, (d) higher/moister range of sampled area, and (e) altitudinal discrimination of biomes in Núi Chúa based on leaf beetle species dissimilarity and their turnover and nestedness components of beta-diversity measured using the framework proposed by [56].
(PDF)

S2 Fig Distance decay analysis of leaf beetle species compositional similarity among sampling points in the Núi Chúa National Park. Data were fitted using least-squares smoothing in R [43]. The proportion of shared species remains consistently low across the geographic distances, with a slight concentration of relatively higher compositional similarities for shorter distances.
(PDF)
