# Supplementary material for: Subtle Ecological Gradient in the Tropics Triggers High Species-Turnover in a Local Geographical Scale
Source: PLoS One. 2016 Jun 8;11(6):e0156840. doi: 10.1371/journal.pone.0156840 (PMC4898766; doi:10.1371/journal.pone.0156840)
Supplement: S1 Fig — Clustering of (a) transects, (b) biomes within forest paths, (c) lower/drier range of sampled area, (d) higher/moister range of sampled area, and (e) altitudinal discrimination of biomes in Núi Chúa based on leaf beetle species dissimilarity and their turnover and nestedness components of beta-diversity measured using the framework proposed by [56]. (PDF) [file pone.0156840.s001.pdf]

SUPPORTING INFORMATION

Subtle ecological gradient in the tropics triggers high species-turnover in a local geographical scale

Dinh T. NGUYEN & Jesús GÓMEZ-ZURITA

S1 Fig. Beta-diversity patterns of leaf beetle communities in Núi Chúa National Park and their decomposition in species replacement and gain/loss (=richness) components.

Clustering of (a) transects, (b) biomes within forest paths, (c) lower/drier range of sampled area, (d) higher/moister range of sampled area, and (e) altitudinal discrimination of biomes in Núi Chúa based on leaf beetle species dissimilarity and their turnover and nestedness components of beta-diversity measured using the framework proposed by [56].

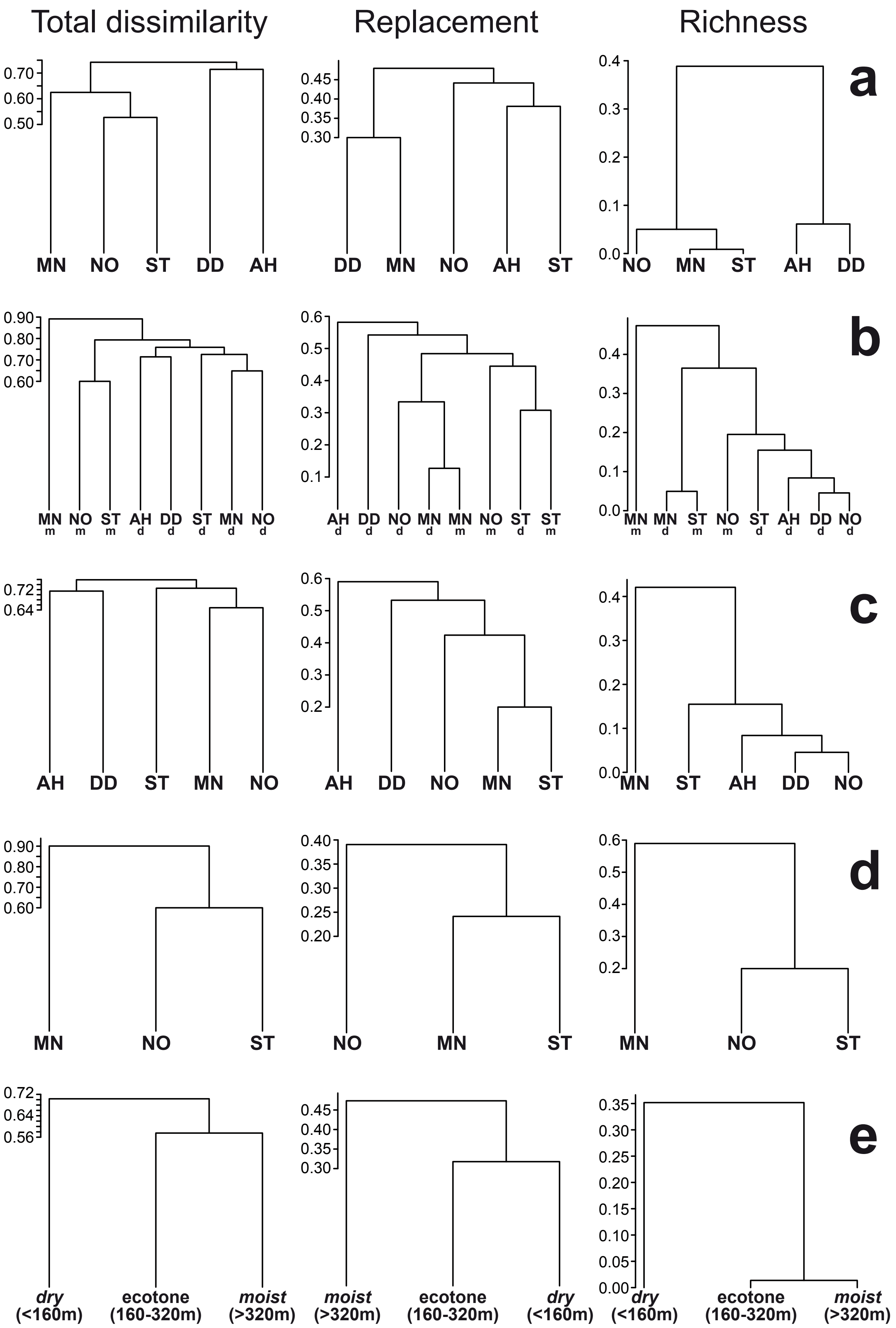

Total dissimilarity
Replacement
Richness
a
b
c
d
e
MN NO ST DD AH
DD MN NO AH ST
NO MN ST AH DD
dry (<160m)
ecotone (160-320m)
moist (>320m)
