# Supplementary material for: Subtle Ecological Gradient in the Tropics Triggers High Species-Turnover in a Local Geographical Scale
Source: PLoS One. 2016 Jun 8;11(6):e0156840. doi: 10.1371/journal.pone.0156840 (PMC4898766; doi:10.1371/journal.pone.0156840)
Supplement: S2 Fig — Data were fitted using least-squares smoothing in R [43]. The proportion of shared species remains consistently low across the geographic distances, with a slight concentration of relatively higher compositional similarities for shorter distances. (PDF) [file pone.0156840.s002.pdf]

SUPPORTING INFORMATION

Subtle ecological gradient in the tropics triggers high species-turnover in a local geographical scale

Dinh T. NGUYEN & Jesús GÓMEZ-ZURITA

S2 Fig. Distance decay analysis of leaf beetle species compositional similarity among sampling points in the Núi Chúa National Park.
Data were fitted using least-squares smoothing in R [43]. The proportion of shared species remains consistently low across the geographic distances, with a slight concentration of relatively higher compositional similarities for shorter distances.

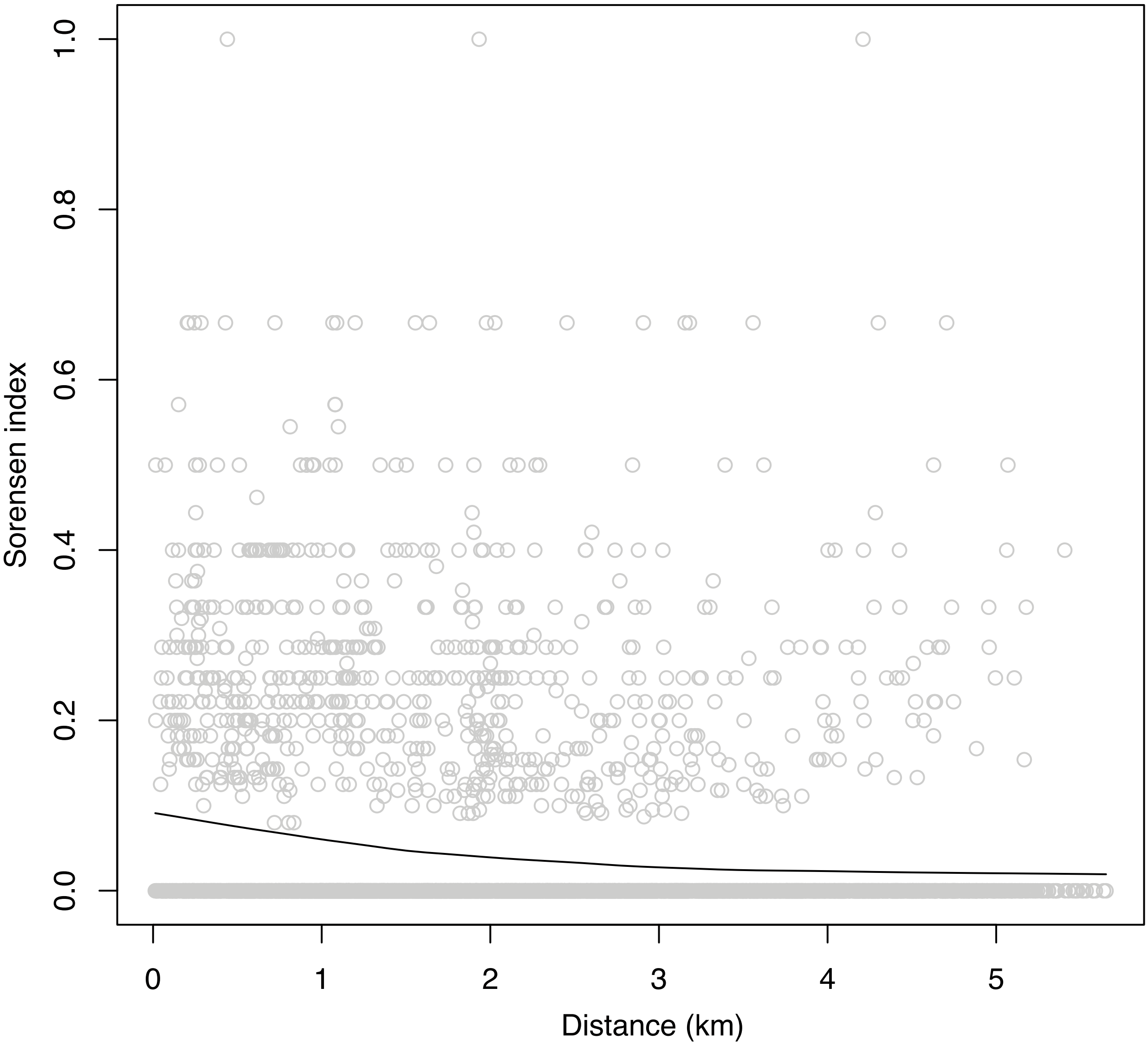
1.0
0.8
0.6
0.4
0.2
0.0
Sorensen index
0
1
2
3
4
5
Distance (km)
